# Genome-wide analysis of the Siboney de Cuba cattle breed: genetic characterization and framing with cattle breeds worldwide

**DOI:** 10.3389/fgene.2024.1302580

**Published:** 2024-01-26

**Authors:** Filippo Cendron, Anel Ledesma-Rodríguez, Salvatore Mastrangelo, Maria Teresa Sardina, Dervel Felipe Díaz-Herrera, Odalys Uffo Reinosa, Martino Cassandro, Mauro Penasa

**Affiliations:** ^1^ Department of Agronomy, Food, Natural Resources, Animals and Environment, University of Padova, Padua, Italy; ^2^ Centro Nacional de Sanidad Agropecuaria (CENSA), Laboratorio de Ensayo para el Control de la Calidad de los Alimentos (CENLAC), San José de las Lajas, Cuba; ^3^ Facultad de Medicina Veterinaria y Zootecnia, Universidad Veracruzana, Xalapa, Mexico; ^4^ Department of Agricultural, Food and Forest Sciences, University of Palermo, Palermo, Italy; ^5^ Faculté des Sciences, Université de Sherbrooke, Sherbrooke, QC, Canada; ^6^ Federazione delle Associazioni Nazionali di Razza e Specie, Roma, Italy

**Keywords:** cattle, genetic diversity, composite breed, copy number variant, Siboney de Cuba

## Abstract

Crossbreeding has been employed to address environmental challenges. One successful example is the Siboney de Cuba, developed in response to economic challenges in the 1960s. The aim of this study was to perform the first genomic characterization of the Siboney de Cuba breed, a successful hybrid breed resulting from the crossbreeding of Cuban Zebu and Holstein, using SNP array chip. For this purpose, 48 Siboney de Cuba cattle samples were collected and genotyped with the GGP Bovine 100k BeadChip, resulting in 83,314 SNPs after quality control. The genetic diversity was investigated using observed and expected heterozygosity, inbreeding coefficient, and minor allele frequency. Runs of homozygosity (ROH) analysis provided insights into molecular inbreeding. Additionally, the study investigated copy number variants (CNV), identifying CNV regions and their distribution. The genetic relationship and population structure of Siboney de Cuba were analyzed in comparison with worldwide cattle populations using ADMIXTURE, multidimensional scaling, and phylogenetic analysis. Six ROH islands containing a total of 50 genes were discovered, some of which were uncharacterized loci. Furthermore, 792 CNV with higher occurrence of genetic material loss were observed. The overall genome coverage for CNV regions was 2.16%. The Siboney de Cuba exhibited a good level of genetic variability with high heterozygosity and low inbreeding when compared with other cattle breeds worldwide. Also, the breed shared genetic similarity to hybrids from America and *Bos indicus* from Africa and highlighted a moderate level of genetic isolation with some overlaps with *Bos taurus* from America. The breed showed a complex genetic composition, influenced by historical factors. Overall, findings of the present study contribute to the understanding of genomic structure of Siboney de Cuba cattle breed.

## 1 Introduction

The domestication of livestock has significantly impacted the social and economic dynamics of numerous human populations worldwide. Through a combination of genetic drift and natural and artificial selection, several breeds have emerged, showcasing a range of phenotypes such as coat color, body size, behavior, and production characteristics (Diamond, 2002; Gautier et al., 2010). Since the advent of the first Industrial Revolution, the genetic diversity of cattle, in Europe and Asia, has been influenced by selective breeding and adaptation to specific environmental conditions. However, there have been instances where the necessity arose to develop crossbreeds that could encounter highly challenging environmental circumstances (Felius et al., 2014; Pitt et al., 2019).

**FIGURE 1:**
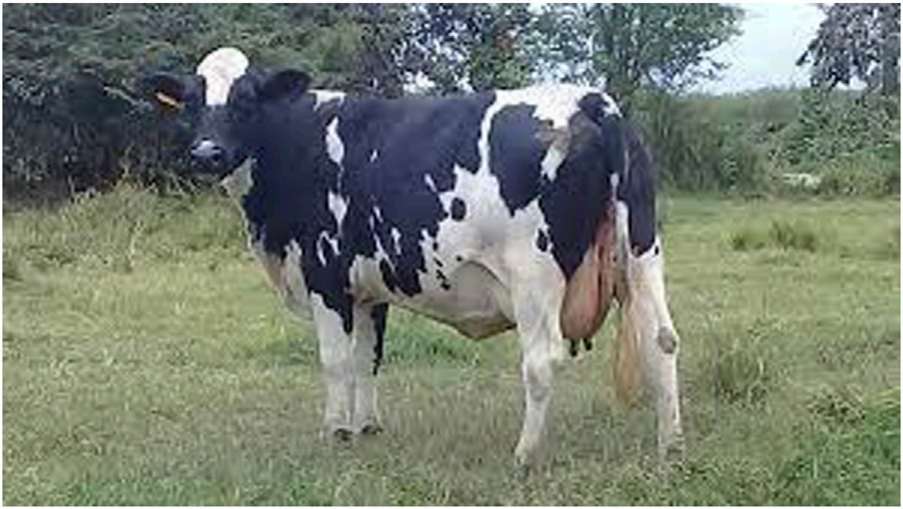
Siboney de Cuba cattle breed.

An example of a hybrid breed that successfully addressed the environmental, economic, and social requirements is the Siboney de Cuba (Figure 1). In the early 1960s, the Cuban cattle population predominantly consisted of Zebu animals (96%), primarily bred for meat production (Ledesma-Rodríguez et al., 2022). However, the economic challenges led to the progressive substitution of the prevailing Zebu population with a breed capable of achieving higher production to meet the increasing demand of milk in Cuba (Acosta et al., 2013).

Through the implementation of artificial insemination, Cuban Zebu was crossed with the most specialized breed for milk production, i.e., Holstein. This resulted in the development of the Siboney de Cuba, which comprises 5/8 Holstein and 3/8 Cuban Zebu (Uffo et al., 2013). The Siboney de Cuba is officially included in the Register of Pure Breeds of Cuba in 1984, with a total of 1,792 males and 68,171 females registered in the herd-book. An official breed standard describes the standard and mandatory identification criteria for the Siboney de Cuba through morphological characteristics to determine whether animals are eligible for registration or not.

The breed demonstrated exceptional adaptability and satisfactory productivity even under extreme climatic conditions (Hernández and Ponce, 2008). The Siboney de Cuba breeding program has been based fundamentally on the improvement of milk production through the identification of the best bulls to be used in the population, without considering other important features such as milk quality, fertility, and longevity.

Several studies have focused on the analysis of productive and reproductive performance of this breed, aiming to identify the best animals for utilization in crossbreeding and genetic improvement programs (Portales et al., 2012; Acosta et al., 2016; García-Díaz et al., 2019). However, to the best of our knowledge, no studies have attempted to assess the genetic structure of the Siboney de Cuba breed using SNP-type molecular markers to provide a comprehensive genetic profile and facilitate direct comparisons with global breeds. The advent of high-throughput SNP genotyping platforms has enabled the implementation of high-density scans using a large number of SNP markers, which can be distributed across the entire genome or concentrated in specific regions in both *Bos taurus* and *Bos indicus*. The SNPs serve as valuable tools to investigate genetic diversity, background, and population structure in livestock and thus evaluate breed biodiversity (McKay et al., 2008; Lin et al., 2010).

The aim of the present study is to explore the diversity and genetic background of the Siboney de Cuba breed, providing the first-ever analysis of its population structure through the SNP array chip. Additionally, this research aims to contextualize the Siboney de Cuba within the global cattle heritage.

## 2 Materials and methods

### 2.1 Samples and genotyping

A total of 48 Siboney de Cuba cattle blood samples (35 females and 13 males, aged between 24 and 30 months) were collected. The DNA was extracted from the blood samples using the phenol–chloroform standard protocol. Animals were chosen on the basis of the viability in the experimental herd of the Centro Nacional de Sanidad Agropecuaria (CENSA, Cuba).

All animals were genotyped using the GGP Bovine 100K BeadChip (*B. taurus* × *B. indicus*) containing approximately 100,000 SNPs (Illumina, San Diego, CA, United States). The genomic coordinates for each marker were obtained using the assembled cow genome (ARS-UCD 1.2). SNPs assigned to sexual chromosomes or without position were discarded. PLINK v. 1.9 software (Chang et al., 2015) was used to perform filtering and quality control on the dataset, using the following criteria: i) minor allele frequency (MAF) ≥ 0.05; ii) genotype call rate for an SNP ≥0.95; and iii) individual call rate ≥0.90. After quality check, 48 animals and 83,314 SNPs were retained.

The raw data were merged with the genotypic data of cattle breeds worldwide to investigate the genetic relationship between Siboney de Cuba and other cattle breeds. The genotypes of 212 breeds were retrieved from Mastrangelo et al. (2020) to create a worldwide dataset. All individuals retrieved from Mastrangelo et al. (2020) were genotyped using the Bovine SNP50K BeadChip (v1 and v2). After the merge, the final dataset consisted of 3,331 animals, 16,102 common SNPs (between the GGP Bovine 100K BeadChip and the worldwide dataset), and 213 populations (divided according to the geographical origin).

### 2.2 Genetic diversity indices

PLINK v1.9 software (Chang et al., 2015) was used to estimate the observed (H_O_) and expected heterozygosity (H_E_), inbreeding coefficient (F_HOM_), and the MAF to compare the results with those reported in the literature (e.g., Decker et al., 2014; Makina et al., 2016; Mastrangelo et al., 2020).

### 2.3 Runs of homozygosity

Runs of homozygosity (ROH) analysis was performed to obtain an estimate of molecular inbreeding using PLINK v1.9 (Chang et al., 2015). To define ROH, the following parameters were fixed: i) the minimum length was set to 1 Mb (–homozig-kb); ii) two missing SNPs and up to one possible heterozygous genotype were allowed in the ROH (–homozyg-window-missing 50 and –homozyg-window-het 1); iii) the minimum number of SNPs that constituted the ROH was set to 50 (–homozyg-snp 50); iv) the minimum SNP density per ROH was set to one SNP every 100 kb (–homozyg-density 100); and v) the maximum gap between consecutive homozygous SNPs was 1,000 kb (–homozyg-gap 1,000). The inbreeding coefficient based on ROH (F_ROH_) for each animal, the mean number of ROH per individual, and the mean length of ROH per individual were estimated. The total percentage of SNPs clustering inside ROH was determined by counting the number of times that each target appeared in ROH and dividing this by the total number of animals (n = 48). To identify regions of high homozygosity, called ROH islands, the top 0.999% of SNPs in the locus homozygosity range was selected. Subsequently, the annotation of gene mapping within ROH islands was also conducted using the list of the cow autosome ARS-UDC 1.2 *B. taurus* from the UCSC Genome Browser database (https://genome.ucsc.edu/).

### 2.4 Identification of copy number variants and regions

Identification of copy number variants (CNV) was performed using PennCNV software (Wang et al., 2007), exploiting the Log R ratio (LRR) and B allele frequency (BAF) (Kumar et al., 2021) obtained from the intensity files of genotyping. The CNV calling at the individual level was carried out through the default parameters of the hidden Markov model: standard deviation of LRR <0.30, BAF drift <0.01, waviness factor value between −0.05 and 0.05, and minimum of 3 SNPs to define CNV. The distribution of CNV per individual spanned from 0 to >100. To avoid the detection of false-positive and/or -negative CNV and outliers, two different “.hmm” (agre.hmm and hh550. hmm) files were used to run PennCNV, as described in Gorla et al. (2017). Indeed, the “.hmm” file may substantially affect the false-positive and false-negative rates. To obtain valid CNV, the common calls from all the hidden Markov models were considered (Gorla et al., 2017). This solved the critical choice about the correct .hmm file to use in order to mapping CNV to control false-positive and -negative calls.

The R package HandyCNV (Zhou et al., 2021) was used on PennCNV output files to summarize CNV and define CNV regions (CNVR). The following package commands were imputed to the analysis: i) *cnv_clean* () function to convert CNV results into a standard format and make basic summary [the CNV larger than 5 Mb were discarded following Pinto et al. (2011)]; ii) *cnv_summarise_plot* () to create the CNV distribution, frequency, and length group plot; and iii) *call_cnvr* () to define CNVR and their frequency (merging CNV that overlapped by at least 1 bp) (Zhou et al., 2022). In the CNVR map and definition, “gain” CNVR indicates the regions that contain more than two copies of CNV, “loss” indicates the regions that contain deleted CNV, and “mixed” indicates the regions that contain at least one duplicated and one deleted CNV. Consensus CNVR were generated with the *call_cnvr* () command by combining the identified CNVR and the overlapping regions in the final CNVR distribution map (Zhou et al., 2022).

### 2.5 Genetic relationship and population structure of the Siboney de Cuba with worldwide cattle populations

The population structure was investigated by applying the model-based clustering algorithm run in ADMIXTURE for K = 2–50 (Alexander and Lange, 2011). The cross-validation procedure was used to estimate the most likely number of populations. The K-value that minimizes the cross-validation prediction error was then assumed as the most likely. The BITE R package was used to graphically represent the results through the membercoef. circos function and estimate the most likely number of clusters following the cross-validation procedure (Milanesi et al., 2017). Pair-wise genetic relationships within and between the breeds were estimated using a matrix of genome-wide identity-by-state genetic distances in PLINK v1.9 (Chang et al., 2015) and plotted using a multidimensional scaling (MDS) plot that represented the components C1 and C2. Phylogenetic relationships between the breeds were analyzed by determining the Reynolds genetic distances using the R package ape (Paradis and Schliep, 2019). Neighbor networks were constructed from the estimated genetic distances using FigTree (Huson and Bryant, 2006). Graphical representation was created using R software (R Core Team, 2020).

## 3 Results

### 3.1 Genetic diversity

Genetic diversity indices were estimated using different approaches in order to identify the levels of variability of the Siboney de Cuba breed. Descriptive statistics are presented in Table 1. Expected and observed heterozygosity showed high levels of diversity (0.404 ± 0.103 and 0.409 ± 0.404, respectively), and F_HOM_ and F_ROH_ highlighted low levels of inbreeding in the population (−0.013 ± 0.033 and 0.004 ± 0.002, respectively).

**TABLE 1:** Mean and standard deviation of genetic diversity indices estimated in the Siboney de Cuba cattle breed.

MAF	H_E_	H_O_	F_HOM_	F_ROH_
0.317 ± 0.120	0.404 ± 0.103	0.409 ± 0.404	−0.013 ± 0.033	0.004 ± 0.002

MAF, minor allele frequency; H_E_, expected heterozygosity; H_O_, observed heterozygosity; F_HOM_, inbreeding coefficient based on excess of homozygosity; F_ROH_, inbreeding coefficient based on runs of homozygosity.

### 3.2 Runs of homozygosity islands

**FIGURE 2:**
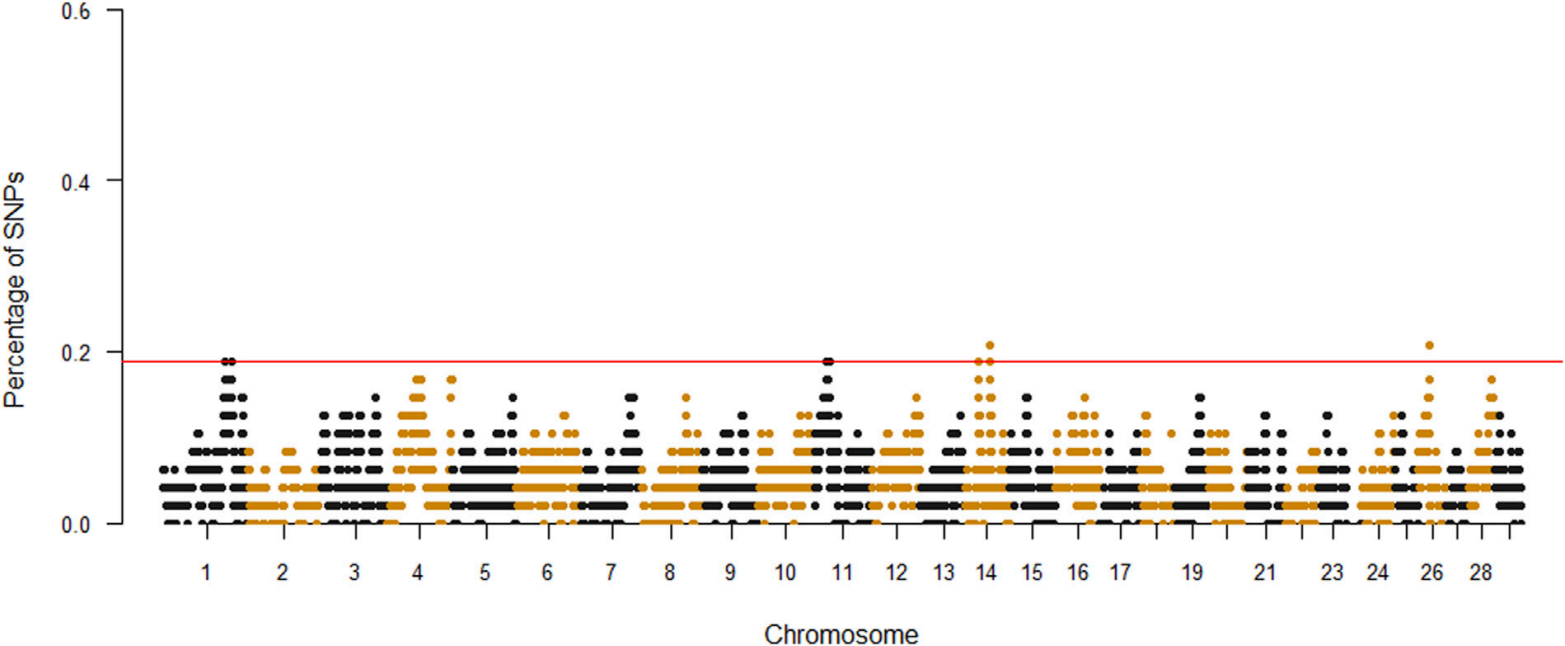
Manhattan plot of each single-nucleotide polymorphism (SNP) significance in runs of homozygosity. The red line indicates the top 0.999% of SNPs.

A total of 1,202 ROH were identified, ranging from 3 to 48 per individual. The average number of ROH per animal was 25.04, with an average length of 4.4 Mb. The top 0.999% SNPs of percentile distribution were selected and used to define genomic ROH islands. In detail, adjacent SNPs over the threshold were merged into the genomic regions corresponding to ROH islands in the Siboney de Cuba (Figure 2). The Manhattan plot showed few peaks above the minimum value, considering each significant marker with a percentage of occurrence >18%.

Table 2 provides a complete description of the identified ROH islands. The chromosome (Chr) position, start and end, and number of SNPs of the islands with the annotated genes were reported. A total of six islands were detected: one in *B. taurus* Chr 1, two in Chr 11 and Chr 14, and one in Chr 26. The islands ranged from 0.03 Mb (Chr 1) to 1.8 Mb (Chr 26). A total of 50 genes were identified, of which 20 were uncharacterized loci (LOC). The region on Chr 26 includes 55 SNPs, while 3 molecular markers are present in Chr 1.

**TABLE 2:** Runs of homozygosity islands identified in the Siboney de Cuba cattle breed.

Chromosome	Start (bp)	End (bp)	SNP (n)	Length (bp)	Gene
1	126,716,497	126,746,747	3	30,250	None
11	23,918,407	25,131,113	47	1,212,706	*LOC113901130*, *LOC113901129*, *PKDCC*, *LOC113901132*, *LOC113901133*, *EML4*, *LOC113901135*, *KCNG3*, *MTA3*, *LOC113902003*, *OXER1*, *HAAO*, *LOC113900702*, and *TRNAI-UAU*
11	26,235,778	27,587,137	50	1,351,359	*PPM1B*, *LOC113900814*, *SLC3A1*, *PREPL*, *LOC113901158*, *LOC113901969*, *CAMKMT*, *TRNAE-UUC*, *LOC113901160*, *SIX3*, *LOC113901161*, *SIX2*, *LOC113901163*, *TRNAC-GCA*, *LOC113901999*, *LOC113900723*, *SRBD1*, and *LOC113901165*
14	24,955,077	25,541,189	30	586,112	*LOC113904194*, *TRNAG-CCC*, *LOC113904515*, *SDCBP*, *NSMAF*, *LOC113904197*, *LOC113903947*, *TOX*, and *TRNAC-GCA*
14	47,385,049	48,145,874	26	760,825	*SLC30A8*, *AARD*, *RAD21*, *UTP23*, *EIF3H*, and *LOC113904528*
26	21,138,161	22,939,258	55	1,801,097	*MIXI1*, *ADD3*, *LOC113884315*, and *XPNPEP1*

The italicized values in Table 2 represent genes.

### 3.3 CNV and CNVR

**FIGURE 3:**
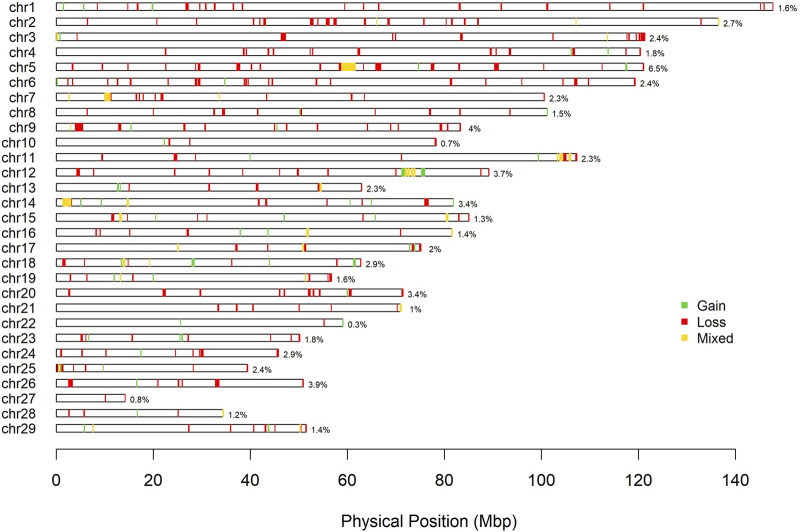
Physical distribution of copy number variant regions on chromosomes according to the state (green: gain; red: loss; yellow: mixed).

A total of 792 CNV were detected in the genome of the Siboney de Cuba (Table 3). The highest number of CNV was associated with the loss of genetic material (type 0 and 1) that covers also the greatest length of the genome. In Table 3, the identified CNVR are also reported, associated with entire length, mean, minimum, and maximum length, and total genome coverage based on the 29 autosomes. A total of 364 CNVR were identified and, as for CNV, the highest number of CNVR was associated with the loss of genomic regions (269), followed by gain (57) and mixed (38). The estimated overall coverage of the genome is 2.16% against the 3.41% found through the CNV. In Figure 3, a summary of CNVR distribution across the 29 chromosomes, divided for each type (loss, gain, and mixed), is reported. A detailed overview of the distribution of CNVR on chromosomes in the studied breed are presented in Supplementary Table S1, where CNVR are divided considering start and end, and the genes inside the regions are annotated.

**TABLE 3:** Descriptive statistics of copy number variants (CNV) and CNV regions in the Siboney de Cuba cattle breed.

CNV						
Type	n	Total length (bp)	Mean (bp)	Minimum (bp)	Maximum (bp)	Genome coverage (%)
**0**	213	21,550,466	101,176	100	413,084	0.84
**1**	374	49,125,222	131,351	100	2,386,977	1.91
**3**	199	16,674,995	83,794	100	812,454	0.65
**4**	6	640,363	106,727	134	444,340	0.02
**Total^a^**	**792**	**87,991,046**	**423,048**	**434**	**4,056,855**	**3.42**

CNVR						
Type	n	Total Length (bp)	Mean (bp)	Minimum (bp)	Maximum (bp)	Genome coverage (%)
**Loss**	269	40,788,349	151,630	100	1,020,336	1.58
**Gain**	57	4,367,383	76,621	100	444,340	0.17
**Mixed**	38	10,480,310	275,798	4,934	2,874,709	0.41
**Total^a^**	**364**	**55,636,042**	**504,049**	**5,134**	**4,339,385**	**2.16**

^a^ Common and not common.

The bold values represent the total values.

### 3.4 Genetic relationship and population structure of the Siboney de Cuba with worldwide cattle populations

**FIGURE 4:**
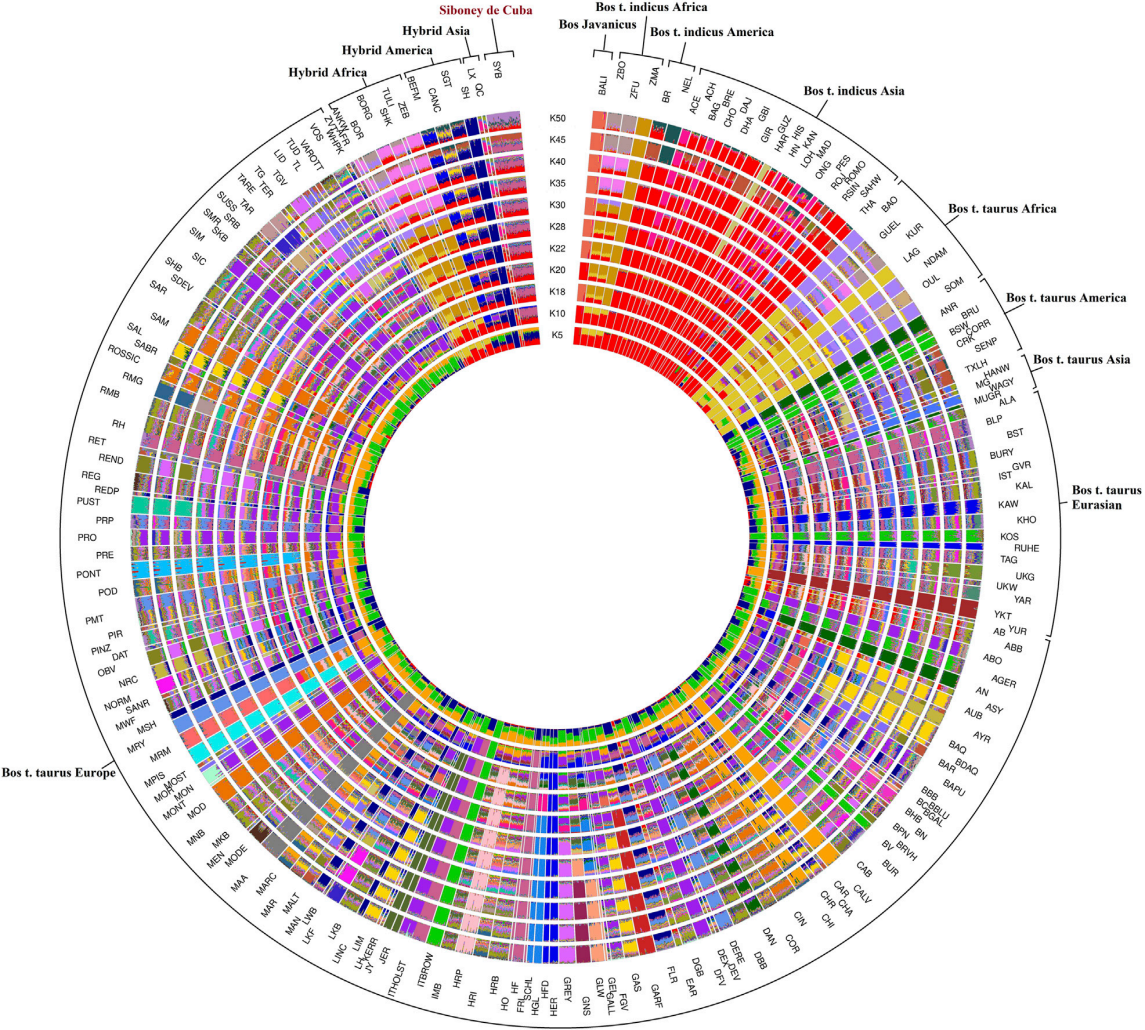
Maximum likelihood estimation calculated using the admixture algorithm. The inferred clusters (K) were reported for K = 2–50. For the full definition of breeds’ acronyms, see Supplementary Table S2 (Mastrangelo et al., 2020).

The admixture analysis, utilizing a range of 2–50 K, provided insights into the population structure in order to identify ancestral components shared among different worldwide bovine populations (Figure 4). To better understand the ancestral component and the genetic relationship, the breeds were clustered considering the geographical distribution. The results at K = 2 highlighted the subdivisions among the domesticated populations by separating *B. taurus* and *B. indicus* cattle. The Siboney de Cuba shares the genetic background with *B. indicus* from Asia, Africa, and America. At K = 12, the genetic background of the Siboney de Cuba shows the marks of *B. indicus* in general, mixed with *B. taurus* from Europe, in particular with Holstein. However, at K = 50, the Siboney de Cuba clustered apart from all the other breeds, with all individuals showing a mixed ancestry and genetic composition.

**FIGURE 5:**
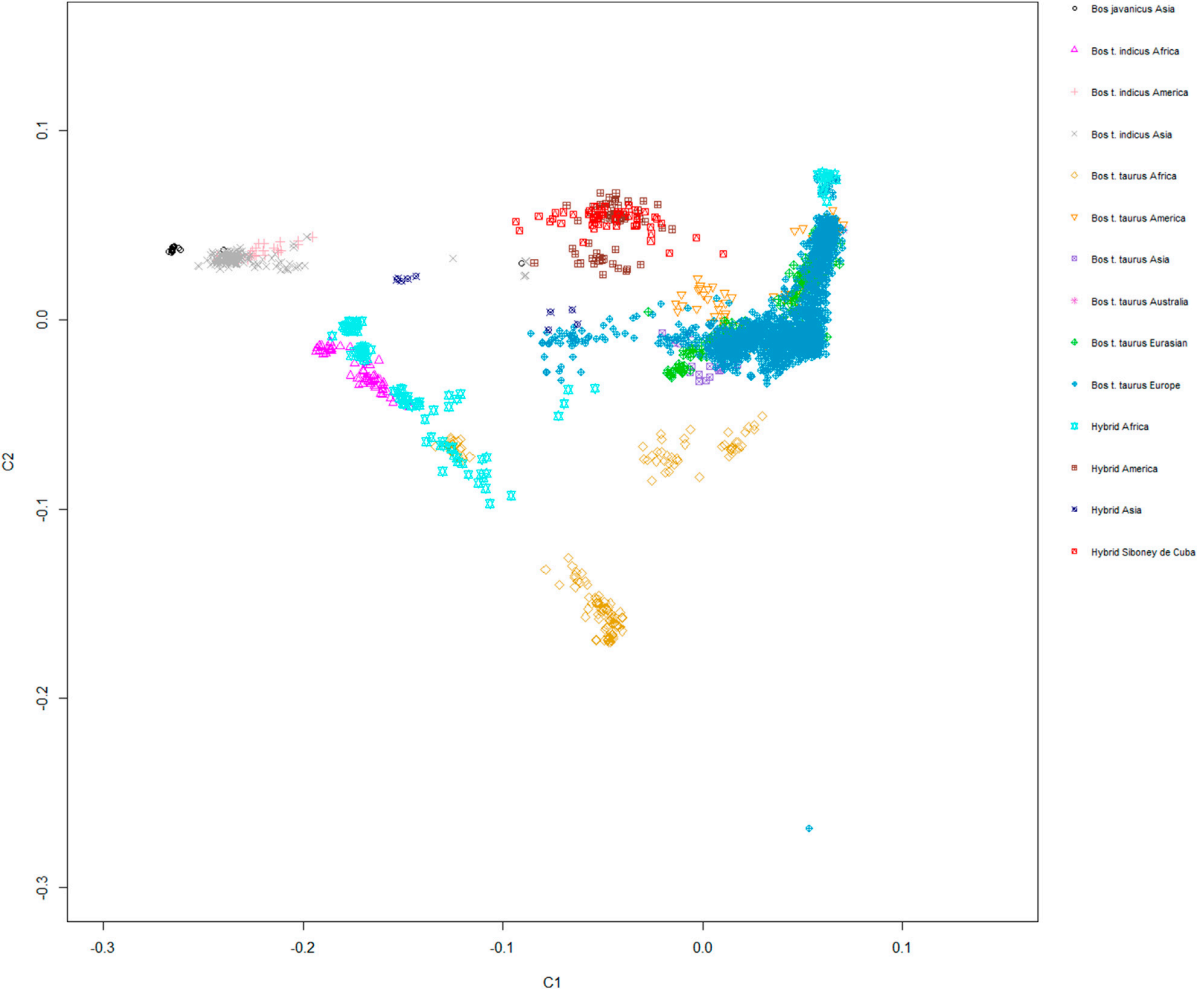
Genetic relationship between the Siboney de Cuba and worldwide cattle breeds as inferred by MDS analysis trough C1 and C2 components. Points are colored according to the geographical origin of breeds.

The MDS plot, graphed for components C1 and C2, showed a moderate level of genetic isolation for the Siboney de Cuba cattle breed, which generated a fairly closed cluster. A stronger overlapping with Santa Gertrudis, Beefmaster, and Canchim breeds, belonging to hybrids from America, is clear (Figure 5).

**FIGURE 6:**
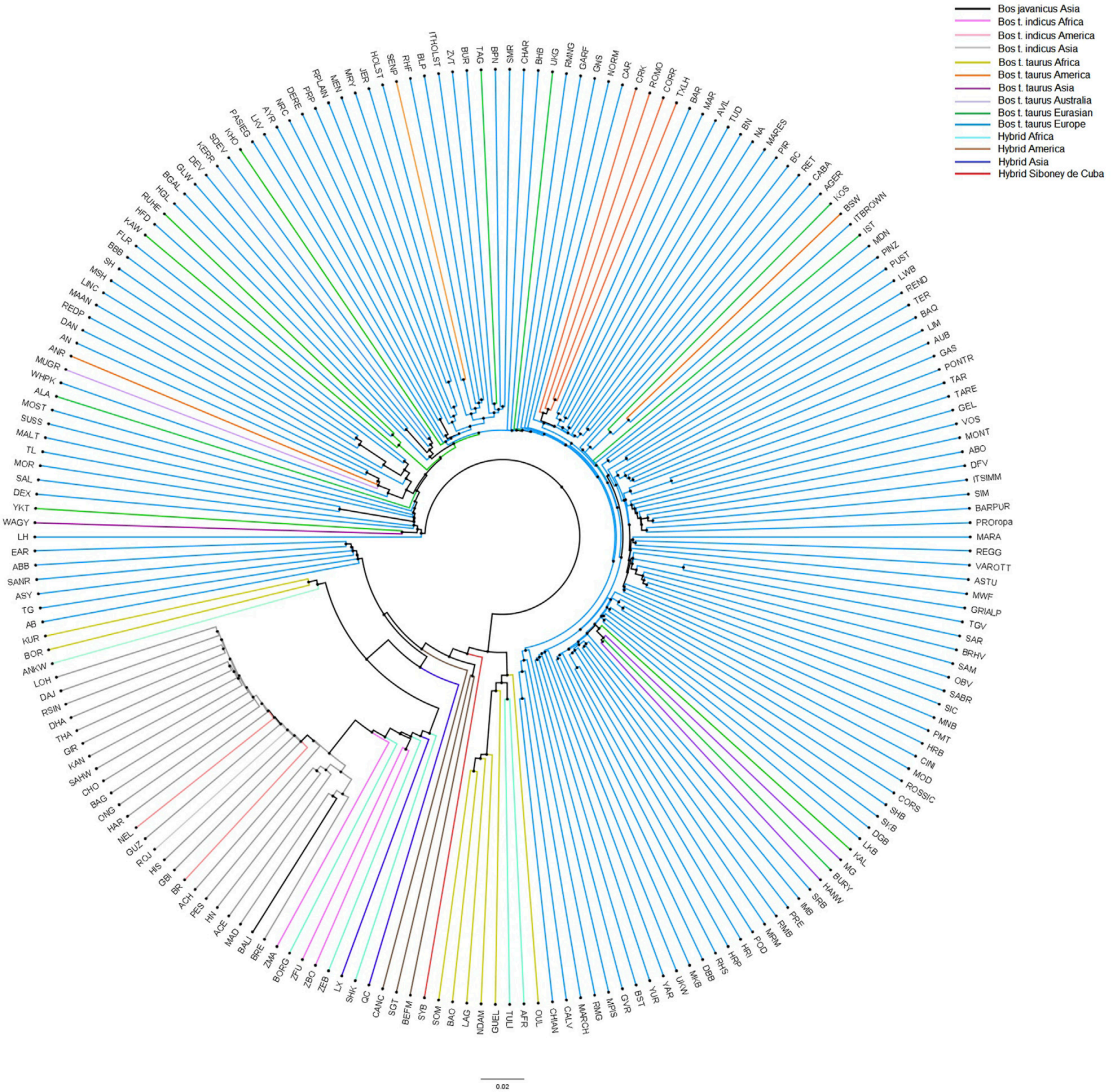
Neighbor-joining tree constructed on the allele sharing distances for the Siboney de Cuba and worldwide breeds. Colors represent the breeds and geographical distribution. For the full definition of breeds’ acronyms, see Supplementary Table S2 (Mastrangelo et al., 2020).

To provide additional insights into the relationships and patterns of divergence, a neighbor-net based on allele sharing distances was constructed (Figure 6). Consistent with the MDS plot, the neighbor-net shows several clusters between the worldwide breeds, with the Siboney de Cuba remaining close to the hybrids from America.

## 4 Discussion

### 4.1 Genetic background of the Siboney de Cuba

The genetic diversity of hybrid populations is an important resource for food security, sustainable rural development, and mitigation of the climate change (Strandén et al., 2019; McIntosh et al., 2023). The ability to create new hybrid breeds that are able to adapt to environmental variations and product demand is important to satisfy human needs. However, hybrid breeds can have different genetic deficits or variations compared to pure or indigenous breeds (McIntosh et al., 2023).

In the present study, a full genetic characterization of the Siboney de Cuba cattle breed is presented. To the best of our knowledge, this is the first study regarding the genomic structure of this breed. In the past, the Siboney de Cuba was genetically characterized by Acosta et al. (2013) through microsatellite molecular markers. The utilization of SNP chips enhances genome characterization compared to microsatellite molecular markers for several reasons. SNP chips offer a higher density of markers across the genome, providing a more comprehensive and detailed view of genetic variations, thus allowing for more precise mapping of genetic traits and a finer resolution in identifying variations associated with specific traits. Furthermore, SNPs are bi-allelic markers, offering a simpler and more straightforward interpretation of genetic data compared to the multi-allelic nature of microsatellite markers, which can be more challenging to analyze accurately (Bovine HapMap Consortium et al., 2009).

The genetic diversity indices (Table 1) underline a good variability associated with the recent history of the Siboney de Cuba (Acosta et al., 2013). However, the estimated H_E_ and H_O_ through SNP showed lower values compared with those reported by Acosta et al. (2013) through microsatellites. The lower values of the indices estimated from SNPs commonly arise because of inherent dissimilarities in marker characteristics. Bi-allelic SNPs generally exhibit reduced allelic diversity than multi-allelic microsatellites, which leads to less heterozygosity. Moreover, factors such as the genomic distribution of SNPs, technical biases, genotyping errors, and variations in sample sizes or studied populations all play a role in the noted discrepancies observed in heterozygosity values (Zimmerman et al., 2020).

The genetic diversity indices presented in the current study are comparable with those reported in previous studies for composite cattle (Marson et al., 2005; Decker et al., 2014) and pure breeds (Engelsma et al., 2012; Senczuk et al., 2020). Makina et al. (2014), in a study on composite breeds (*B. taurus* x *B. indicus*), reported lower genetic diversity indices compared to the Siboney de Cuba, with lower H_E_, which suggests that the Siboney de Cuba has maintained a higher level of genetic diversity, likely because of its more recent history.

Considering that Siboney de Cuba cattle is a hybrid breed with genetic characteristics from both *B. taurus* and *B. indicus*, it was anticipated that the average MAF would be higher when including indicine-derived SNPs. In this study, the average MAF (0.317) was higher than the MAF of composite breeds reported by Edea et al. (2015). The utilization of indicine-derived SNPs appeared to reduce the ascertainment bias, as the GGP Bovine BeadChip was intentionally developed to incorporate a greater number of SNPs of indicine origin, as reported in Edea et al. (2020) and Ferraz et al. (2020).

Monitoring and controlling inbreeding is important to limit the potential impact of deleterious alleles, inbreeding depression, and loss of genetic variation. Currently, the method based on ROH is considered one of the most powerful approaches to estimate the genomic inbreeding (Mastrangelo et al., 2017). The genomic inbreeding showed a relevant low homozygosity in accordance with Acosta et al. (2013). The maintenance of low levels of inbreeding is also desirable in composite breeds, as an advantage of crossbreeding is heterosis.

Overall, the results indicate a strong genetic variability for Siboney de Cuba cattle, which corroborates the findings of Uffo et al. (2013) on the same breed through microsatellites. These outcomes are useful when considering the possibility of response of this breed when submitted to genetic selection programs. Therefore, the current study on the genomic characterization of the Siboney de Cuba breed provides feedback about successful efforts to keep the inbreeding level within the population low.

The genomic locations of ROH are important genomic footprints of information on the demographic history of livestock species (Bosse et al., 2012). 

ROH islands might be indicative of genomic regions that underwent natural and/or artificial selection. Five out of the six islands allowed us to identify different known genes potentially under selection. Several uncharacterized *LOC* genes were observed, reflecting the selection action on uncharacterized regulatory regions or simply the fixation of non-coding DNA by genetic drift due to a mild selection (Bosse et al., 2012). In contrast, several coding genes are located inside the ROH islands. Some of these genes are worth mentioning because they show a particular relationship with the productive traits. The *PPM1B* (*protein tyrosine phosphatase*, Mg^2+^/Mn^2+^-dependent 1B) gene on Chr 11 was negatively associated with muscle atrophy; indeed, *PPM1B* expression gradually decreases when muscle atrophy increases (Wei and Liang, 2012). On Chr 14, the *NSMAF* (*neutral sphingomyelinase activation-associated factor*) gene seems to be involved in cattle growth, development, feed efficiency, and carcass traits in the Red Angus breed (Smith et al., 2022). The *TOX* (*thymocyte selection-associated high mobility group box*) gene acts as a transcription factor in the hypothalamus and plays a key role in the development of puberty in Brahman cattle (Fortes et al., 2012). Causal variants of *TOX* were associated with reproductive traits in Nellore cattle (De Camargo et al., 2015); however, it seems to play a role in the quality of the carcass traits in Korean Hanwoo cattle (Bhuiyan et al., 2018).

The *RAD21* (*RAD21 cohesin complex component*) gene was also identified in the Siboney de Cuba and its results were associated with carcass weight and eye muscle area development in cattle. The gene is located on Chr 14 and belongs to the sixth island in ROH analysis. Inside the last region identified, the *ADD3* (*adducin 3*) gene on Chr 26 was identified; it is related to the development and growth traits in cattle (Huang et al., 2019). It is worth noting that the presented analysis also found an interesting gene on Chr 26, *XPNPEP1*, (*X-prolyl aminopeptidase 1*) related to milk production traits (Ibeagha-Awemu et al., 2016). In detail, Ibeagha-Awemu et al. (2016) conducted GWAS and found that this gene is related to the presence of tridecylic acid (C13:0) in milk.

### 4.2 CNV and CNVR diversity in the Siboney de Cuba

Considering the growing body of evidence across diverse mammalian species, which suggests that CNV have the capacity to directly influence phenotypic variation, our objective was to conduct a comprehensive investigation of CNV prevalence in Siboney de Cuba cattle (Keel et al., 2016). This study represents the first CNV scan in the Siboney de Cuba utilizing a medium-density SNP chip, offering valuable insights into structural genomic variations that contribute to the enhancement of the genome Bovine CNV map. A major finding is that the total proportion of the genome spanned by CNV is lower (3.41%) in the Siboney de Cuba compared with other cattle breeds such as Valdostana, Holstein-Friesian, and Xin Jiang Brown (Durán Aguilar et al., 2017; Strillacci et al., 2018; Zhou et al., 2022).

A lower count of duplications (gain state) than deletions (loss state) in the Siboney de Cuba were observed. This finding corroborates previous reports based on SNP analyses (Jiang et al., 2013) and whole-genome sequencing studies (Gao et al., 2017), indicating the presence of substantial genetic variability in the bovine breed under investigation. Some of the candidate genes identified in ROH islands are also found in CNVR, such as *PKDCC* and *EML4* in CNVR 176 and *SLC3A1* in CNVR 281 (Supplementary Table S1). This supports the possibility of major selection pressure on these genes; however, their function is associated with several marginal biological processes not related with quantitative/qualitative traits. For instance, the gene *PKCDD* (*protein kinase domain-containing*, *cytoplasmic*) seems to be involved in peptidyl-tyrosine phosphorylation and skeletal system development (Vitorino et al., 2015). *EML4* (*echinoderm microtubule-associated protein-like 4*) is a member of the echinoderm microtubule-associated protein-like family conserved among different species as the mouse, monkey, human, and cattle (Sabir et al., 2017). Finally, the gene *SLC3A1* (*solute carrier family 3 member 1*) provides instructions for producing one part (subunit) of a protein made primarily in the kidneys. During the process of urine formation in the kidneys, this protein complex absorbs particular protein building blocks (amino acids) back into the blood. In particular, the amino acids such as cysteine, ornithine, arginine, and lysine are absorbed back into the blood through the mechanism in which the encoded protein is involved (Calonge et al., 1995).

### 4.3 Genetic comparison between Siboney de Cuba and worldwide breeds in relation with the geographical distribution

Population structure analysis allowed us to distinguish the Siboney de Cuba from other cattle populations. The MDS plot (Figure 5) shows a strong overlap with the breeds of the cluster of American hybrids and proximity with *B. taurus* from America. This result was expected because of the historical and political situation in Cuba (Portes, 2007). The presence of an embargo has probably limited the possibility of developing a genetic reservoir composed by animals from other parts of the world (Carucci, 2000). The genetic situation of the Siboney de Cuba is confirmed by the phylogenetic tree that corroborates the results from the MDS plot. Curiously, the influence of *B. taurus* from Africa is clear in the phylogenetic tree. However, components C1 and C2 of the MDS plot classify separately the African populations from the Siboney de Cuba. Probably, some genetic traces of *B. taurus* from Africa may be present in the Siboney de Cuba due to the events during the 17th century (Guerra, 2020).

Admixture analysis corroborates the results from the MDS plot; however, it also shows that *B. indicus* from America seems to contribute to the genetic background of the breed under investigation as well as the Holstein from Europe. Although the results from admixture analysis confirm the cluster analysis obtained through MDS, the genetic background of the Cuban hybrid breed is confused.

As described by Mastrangelo et al. (2020), some hybrid populations from America showed a close correlation with the European breeds. The presence of European Holstein genetic ancestry components in American Holstein cattle and their integration into the genome of the Siboney de Cuba suggests the potential for migratory flow or commercial exchange between Europe and America. This phenomenon highlights the historical interactions and genetic exchanges that have influenced the genetic composition of Siboney de Cuba cattle; indeed, due to the Cuban embargo restricting the importation of Holstein cattle from America and considering that these cattle comprise genetic elements of European origin, their traces persist within the genome of Siboney de Cuba. It is worth noting that a study of migration flows could account for most of these findings, as reported by Scheu et al. (2015).

Interestingly, as described in Acosta et al. (2013), the Siboney de Cuba has a strong correlation with the other breeds, both local and hybrid, from the Cuban cattle population, highlighting a close system of breeding, confined to the island. Although this aspect was expected, the results showed that the animals belonging to the group of American hybrids strongly overlap with the Siboney de Cuba; however, admixture analysis showed that the genetic background differs between these populations. In this way, the Siboney de Cuba is far from the breeds of the European and Asiatic context, and it seems to have a closer genetic relationship with the breeds from Africa and America.

## 5 Conclusion

Most of the endeavors aimed at enhancing the genetic potential of the Siboney de Cuba have traditionally relied on conventional approaches. In the present paper, the genomic characterization of the Siboney de Cuba through SNP molecular markers was performed. Nonetheless, the application of genomic techniques will have a positive impact on the genetic improvement of the breed as it will contribute to reduce the generation interval and inbreeding. Undoubtedly, genomic selection is one of the crucial tools that the Cuban livestock industry needs to fully exploit the desired characteristics of the breed. By harnessing genomic data, it is possible to strategically design breeding programs aimed at selecting the individuals which can contribute to improve the traits of interest for the industry. Moreover, the insights garnered from genomic characterization allow the implementation of measures to monitor and control the inbreeding levels in the population. Therefore, the application of genomic techniques not only contributes to better characterize the Siboney de Cuba breed but also has the potential to effectively contribute to the enhancement of traits of economic relevance in the Cuban livestock industry.

## Data Availability

The data presented in the study are deposited in the Research Data Unipd repository, Item ID 1057 (https://researchdata.cab.unipd.it/1057).
